# Travel Time to Health Facilities as a Marker of Geographical Accessibility Across Heterogeneous Land Coverage in Peru

**DOI:** 10.3389/fpubh.2020.00498

**Published:** 2020-09-16

**Authors:** Gabriel Carrasco-Escobar, Edgar Manrique, Kelly Tello-Lizarraga, J. Jaime Miranda

**Affiliations:** ^1^Health Innovation Laboratory, Institute of Tropical Medicine “Alexander von Humboldt, ” Universidad Peruana Cayetano Heredia, Lima, Peru; ^2^Division of Infectious Diseases, Department of Medicine, University of California, San Diego, La Jolla, CA, United States; ^3^Facultad de Salud Publica y Administración, Universidad Peruana Cayetano Heredia, Lima, Peru; ^4^CRONICAS Centre of Excellence in Chronic Diseases, Universidad Peruana Cayetano Heredia, Lima, Peru; ^5^School of Medicine, Universidad Peruana Cayetano Heredia, Lima, Peru

**Keywords:** travel time, geographic accessibility, health care accesibility, spatial analysis, inequality, healthcare delivery

## Abstract

To better estimate the travel time to the most proximate health care facility (HCF) and determine differences across heterogeneous land coverage types, this study explored the use of a novel cloud-based geospatial modeling approach. Geospatial data of 145,134 cities and villages and 8,067 HCF were gathered with land coverage types, roads and river networks, and digital elevation data to produce high-resolution (30 m) estimates of travel time to HCFs across Peru. This study estimated important variations in travel time to HCFs between urban and rural settings and major land coverage types in Peru. The median travel time to primary, secondary, and tertiary HCFs was 1.9-, 2.3-, and 2.2-fold higher in rural than urban settings, respectively. This study provides a new methodology to estimate the travel time to HCFs as a tool to enhance the understanding and characterization of the profiles of accessibility to HCFs in low- and middle-income countries.

## Introduction

Despite growing consensus to combat inequalities in accessibility to health care around the world, large disparities in health care accessibility remain a problem in countries with an ongoing rural-to-urban transition. According to the “Tracking Universal Health Coverage: 2017 Global Monitoring Report,” half of the worldwide population lacks essential health services (1). To overcome the disadvantage of marginalized populations, the international community through the United Nations (UN) has stated 17 Sustainable Development Goals (SDG) that are being targeted by 2030 (2). From these goals, the interface between goal 3— “Good health and well-being” and goal 10— “Reduced inequalities” plays an important role to foster and couple endeavors toward ensured access to health care services.

Health care access focuses on multiple domains such as the provision of health care facilities, supply chain, quality and effective services, human resources, and on the demand side, health-seeking behaviors (3–5). All these characteristics point to the ability of a population to receive appropriate, affordable, and quality medical care when needed (6). In accordance with Tudor's inverse health care law (7), the most common factors that prevent access to health care in rural and high poverty areas are geographical accessibility, availability of the right type of care, financial accessibility, and acceptability of service (4, 8). This study focuses on the travel time to health facilities as an important component of the geographical (or physical) accessibility to health care.

Several studies in developing countries report that geographical accessibility is the main factor that prevents the use of primary health care access (6, 8–12) and not only conditions the ability of the population for health-seeking but also the capacity of the health system to implement prevention and control strategies with adequate coverage. However, fewer studies have explored the heterogeneity in geographical accessibility across areas with contrasting land coverage (13, 14), i.e., the marked variation in the topography and environment conditions overlapped with different transport facilities between rural and urban areas that may influence the geographical accessibility across these areas. The geographical accessibility to health services and health care facilities (HCF) has a direct impact on health outcomes since it determines the timeliness of the response to patients that seek care, community-based campaigns (i.e., vaccination, iron supplements to combat anemia, etc.), or the delivery of first response to accidents or natural disasters.

Previous studies highlighted the importance of geographical or physical accessibility using a variety of methods (14–17). The emergence of “Precision Public Health” driven by estimates of a wide range of health indicators at a high spatial resolution is defined as the use of the best available data to target more effectively and efficiently interventions of all kinds to those most in need (18–21). This approach may be favorable since traditionally government reports aggregate data at administrative units, in a way that obscures the prioritization of resources. A recent study used a precision public health approach to estimate the geographical accessibility to major cities (22), and recently for estimating the geographical accessibility to health facilities in developing countries (23).

This study sought to estimate the travel time to the most proximate health facility in rural and urban areas across heterogeneous land coverage types in Peru as a means to help resource prioritization, disease surveillance, as well as prevention and control strategies. Multiple sources of geospatial data were fitted with a novel cloud-based geospatial modeling approach (22), using the Google Earth Engine platform, to produce high-resolution (30 m) estimates of travel time to the most proximate health facility across the country. These estimates were then compared between urban and rural settings and across 16 major land coverage types in Peru.

## Methods

### Study Design

This is an ecological study using the Peruvian registry of villages and health facilities to model the travel time required for individuals in each village to reach the most proximate health facility (shortest travel time) in a two-step process. First, a friction surface was computed. Several geospatial datasets (land coverage types, boundaries of restricted areas, roads infrastructure, navigable river networks, and topography) were used to construct a surface (i.e., raster or grid), as it was constructed in previous studies (22, 24, 25), of a given spatial resolution (i.e., 30 m per pixel) where the value of each pixel (or cell) contains the time required to travel one meter in that given area. Secondly, this friction surface and the geolocation of the health facilities were used to infer the travel time to the most proximate (shortest travel time) health facility using a cumulative cost function. The shortest travel time was computed based on the speed at which a person can move through different types of land cover and infrastructure, using different types of transportation (i.e., road infrastructure uses motorized vehicles as default and land cover types uses walking speeds as default) (Supplementary Information 1). As a result, the travel time estimate for the most proximate health facility was computed for the entire country at a 500-m spatial resolution. The computed values were summarized per district, province, or department; by urban/rural areas; and across 16 major land coverage types defined by the Ministry of Environment (MEnv).

### Study Area

**Figure 1:**
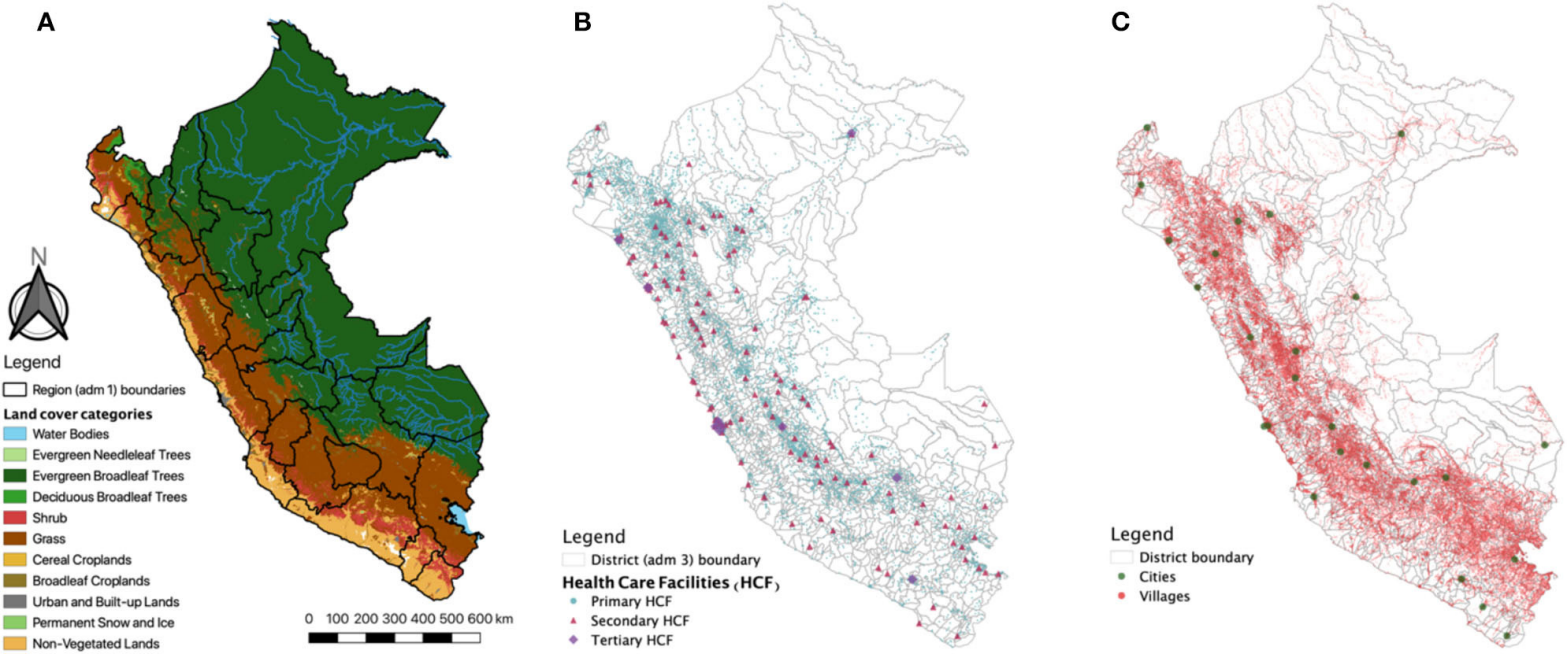
Study area. **(A)** Land coverage and major ecological areas (Coast, Andes, and Jungle) in Peru. Solid black lines represent the 25 Departments (administrative level 1). **(B)** Spatial location of primary, secondary, and tertiary health care facilities (HCF). **(C)** Spatial location of villages and cities. Maps were produced using QGIS, and the base map was derived from satellite images from MODIS MCD12Q1 product.

This study was conducted using nationwide data from Peru, located on the Pacific coast of South America. Peru encompasses an area of 1,285,216 km^2^ and 32,162,184 inhabitants divided in 25 departments and 1,722 districts. Major ecological areas in the country are divided into the coast, highlands, and jungle (Figure 1A); however, this study explores a higher granularity of ecological areas with more than 60 unique land coverage areas (Supplementary Information 2) that were officially classified in Peru. This classification was based on ecological, topographic, and climate characteristics that in turn are important for the calculation of travel time since each land cover type requires a different displacement effort.

### Data Sources

The datasets were divided according to their use in the construction of the friction surface and the travel time map.

### Increase Indentation of Friction Surface Construction

The land coverage types were used from MODIS MCD12Q1 Land Cover type 1 product from 2017 (26). The MODIS collection includes 17 land coverage types including urban and rural areas inferred by the spectral signature of the satellite images at 500-m spatial resolution. The boundaries of the national protected natural areas were included using vector data provided by the MEnv from 2014. The road infrastructure in all districts were provided in vector format by the Peruvian Ministry of Transportation (MTrans), and the navigable river network was derived from the HydroSHEDS Flow Accumulation dataset (27), from the year 2000 at 15 Arc-Seconds spatial resolution (~455 m at a latitude of −10°). The navigable rivers were defined as rivers that have a discharge > 125 cubic meters and were filtered in Google Earth Engine (GEE) (28) using the function *ee.Image.gt*. Permanent water bodies i.e., lakes and lagoons are included in the land cover dataset, a full list of categories of the land cover and the other datasets, as well as associated speed and transportation mode can be found in the Supplementary Information 1. The estimates of the friction surface (minutes required to travel 1 m) were created using the land cover, road infrastructure, and navigable rivers datasets and adjusted by the elevation and slope of the terrain. This means that the travel time required to cross an area will be proportionally dependent on the elevation and slope of the terrain. The elevation data for each area was derived from the SRTM Digital Elevation Data (29) produced by NASA from 2000 at 30 m spatial resolution and the slope at each pixel was calculated from it using the *ee.Terrain.slope* function in GEE. In addition, the national protected natural areas were used to penalize the speed of the land cover datasets to avoid the algorithm going into these areas but also addressing the possibility of persons traveling from those areas to a health facility, as many of them are inhabited by local populations.

### Increase Indentation of Travel Time Estimation

This analysis was conducted only for HCFs of the Ministry of Health (MH) or Regional Governments (RG) that together comprises the public regime of health welfare in Peru. The health care system in Peru was described elsewhere (30); overall it is a complex system with overlapping providers of services and insurance. The HCF from the MH and RG provides health services for 60% of the population; however, in rural areas, they are the only health care providers. The geo-localization of these HCFs was obtained from the national registry of health care facilities (RENAES in Spanish, updated up to 2017) (Figure 1B) and used as target locations for the cumulative cost function. The MH organizes the HCF in three categories according to the complexity of services they provide (from primary health care to specialized hospitals). Primary HCF includes basic health facilities with no laboratory, the secondary HCF includes health facilities with laboratory (including maternities), and the tertiary HCF includes hospitals and higher complexity services. Finally, travel time estimates were extracted for each city and village (Figure 1C). The most updated geo-localization of villages was provided by the Ministry of Education (MEd) in a recent census of cities and villages and education facilities (updated up to 2018).

### Data Analysis

#### Friction Surface Construction

The estimation of travel time was conducted in Google Earth Engine (GEE) (28). A grid surface was constructed using the information about land coverage, road infrastructure, and river network. All datasets were converted into aligned grids with a 30-meter resolution, raster datasets were reclassified changing the values of the categories with the corresponding values of speed (Supplementary Information 1) using the function *ee.Image.remap* in GEE, and vector datasets were converted into rasters using the *ee.Image(0).byte().paint()* function. Each dataset contained information on the speed of movement in each feature. All the layers were then combined in a single *ImageCollection* object in GEE and reduced to an *Image*, with the fastest mode of movement taking precedence (km h^−1^), i.e., using the *ee.ImageCollection.max()* function. The speed assigned for each category of land cover was obtained from elsewhere (22), and the speed for road infrastructure was obtained from the MTrans. A data transformation was conducted, so each pixel within the 2D grid contained the cost (time) to move through the area encompassed in the pixel (the grid surface previously contained speed values in km h^−1^), herein referred to as “friction surface.” The elevation and slope adjustments were carried out using the Elevation adjustment factor and the Slope adjustment factor (based on Tobler's Hiking Function) (Equations 1 and 2) (31), respectively, as used in Weiss, et. al. [22). The speed was penalized (reduced) in urban and national protected areas to account for vehicular traffic and restricted displacement, respectively.

(1) $$\begin{array}{r} Elevation\;adjustment\;factor=1.016e^{-0.0001072\;x\;elevation} \end{array}$$

(2) $$\begin{array}{r} Slope\;adjustment\;factor=6e^{-\frac{3.5}{\tan\left(0.01745\;x\;slope\;angle\right)+0.05}}/5 \end{array}$$

The friction surface combines different types of land cover, road infrastructure, and river networks; therefore, the travel speeds (and cost time) while moving in them correspond to different kinds of transportation (i.e., walking, motorized, boat). The travel scenario for each of the datasets used in the friction surface are listed and described in Supplementary Information 1 and the GEE code for the construction of the friction surface is available in Supplementary Information 3.

#### Travel Time Estimation

To calculate the travel time from the villages to the most proximate health facility, the *ee.Image.cumulativeCost* (cumulative cost) function was used in GEE to generate the accessibility map. The cumulative cost function is a least-cost-path algorithm; briefly, all possible paths were analyzed iteratively and the weighted cost (in this case, weighted by time) was then minimized. The minimum travel time to the most proximate health facility was computed for each pixel in the grid at a 500-m resolution (Supplementary Information 3). To average the travel time at the district level, values were truncated between the 5% and 95% percentile range to avoid extreme values. Since a health facility could be located in the 30 m^2^ corresponding to the pixel spatial resolution of the estimates, a baseline 10-min travel time was considered. The analysis was carried out for each HCF category. After GEE processing, all data outputs were imported and analyzed using R software v.4.0.2 [R Core Team (2020). R: A language and environment for statistical computing. R Foundation for Statistical Computing, Vienna, Austria. URL https://www.R-project.org].

The computed travel time was then summarized per district, province, or department; by urban/rural areas; and across 16 major land coverage types defined by the MEnv. Urban/rural status was defined based on the MODIS land coverage satellite images (described previously in 2.3 Data Sources). To better detail, the large diversity of land coverage types in Peru, a shortlist of 16 eco-regions provided by the MEnv (Supplementary Information 2) was used to summarize the travel time in these areas.

#### Socio-Economic and Epidemiological Metrics

We explored the trends of travel time to HCF in relation to four socio-economic and epidemiological metrics at the district-level (*n* = 1,874). First, the proportion of the population with at least one unsatisfied basic needs (UBN)—a multidimensional poverty measurement developed by the United Nation's Economic Commission for Latin America and the Caribbean (ECLAC). The proportion of the population with at least one UBN was provided by the Ministry of Economy (MEco) and was based on the following indicators: (1) dwelling, (2) sewerage, (3) education, and (4) income. Second, the poverty proportion was provided by the MEco following the small-area estimation methodology (32, 33). Third, the pneumonia fatality rate per 100 cases in children under 5 years was provided by the MH. Finally, the anemia prevalence in children between 6 months and 5 years old followed World Health Organization (WHO) standards (34). Trends between the aforementioned metrics and travel time to HCF were explored using estimates and 95% confidence intervals based on a locally estimated scatterplot smoothing (loess) regression with a span of 0.75.

## Results

### Travel Time to Health Facilities

For this study, we gathered geo-referenced data on 145,134 villages (Figure 1B) and 8,067 HCFs (primary HCFs: 7881, secondary HCFs: 141, and tertiary HCFs: 34) across the 1,722 districts (Figure 1C) in the Peruvian territory. The health facility density (number of health facilities divided by the total population) in Peru was 2.51 per 10,000 inhabitants with variations between major ecological areas, from 1.35 in the coast, 4.56 in the highlands, to 5.21 in the jungle.

**Figure 2:**
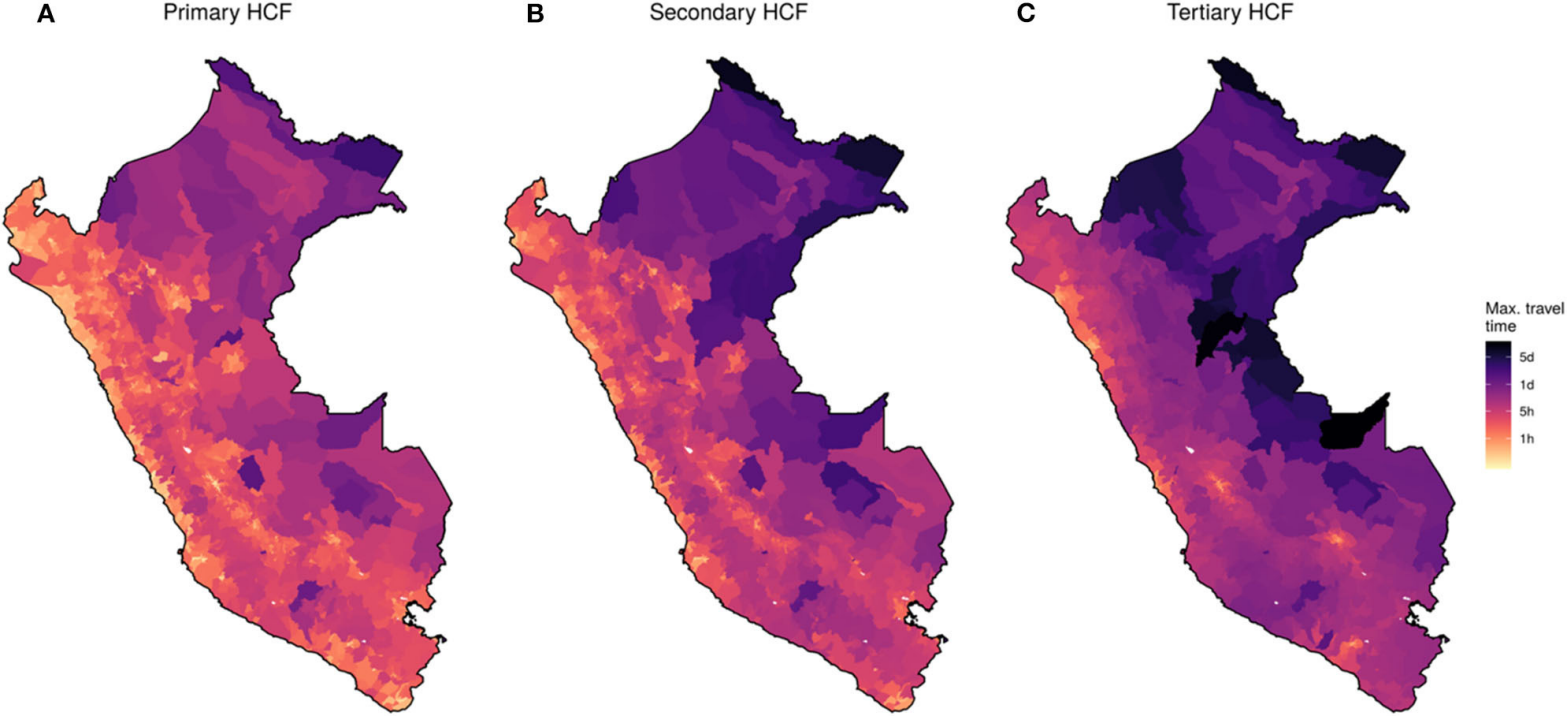
Country-wide map of travel time to health facilities for 2018. District-level maximum travel time to each category of health care facilities (HCF). **(A)** Primary, **(B)** Secondary, and **(C)** Tertiary HCF. Color scale in logarithmic scale.

Friction and travel time maps were reconstructed in the Google Earth Engine using the described local datasets at a spatial resolution of 30 meters per pixel (Supplementary Information 3). Country-wide median travel time from each village to the most proximate HCF varies according to category: primary HCF = 39 min (IQR = 20–93), secondary HCF = 152 min (IQR = 75–251), and tertiary HCF = 448 min (IQR = 302–631). Importantly, maximum travel time reached 7,819, 12,429, and 35,753 min for primary, secondary, and tertiary HCF, respectively (Figure 2).

### Urban/Rural and Ecological Settings

**Figure 3:**
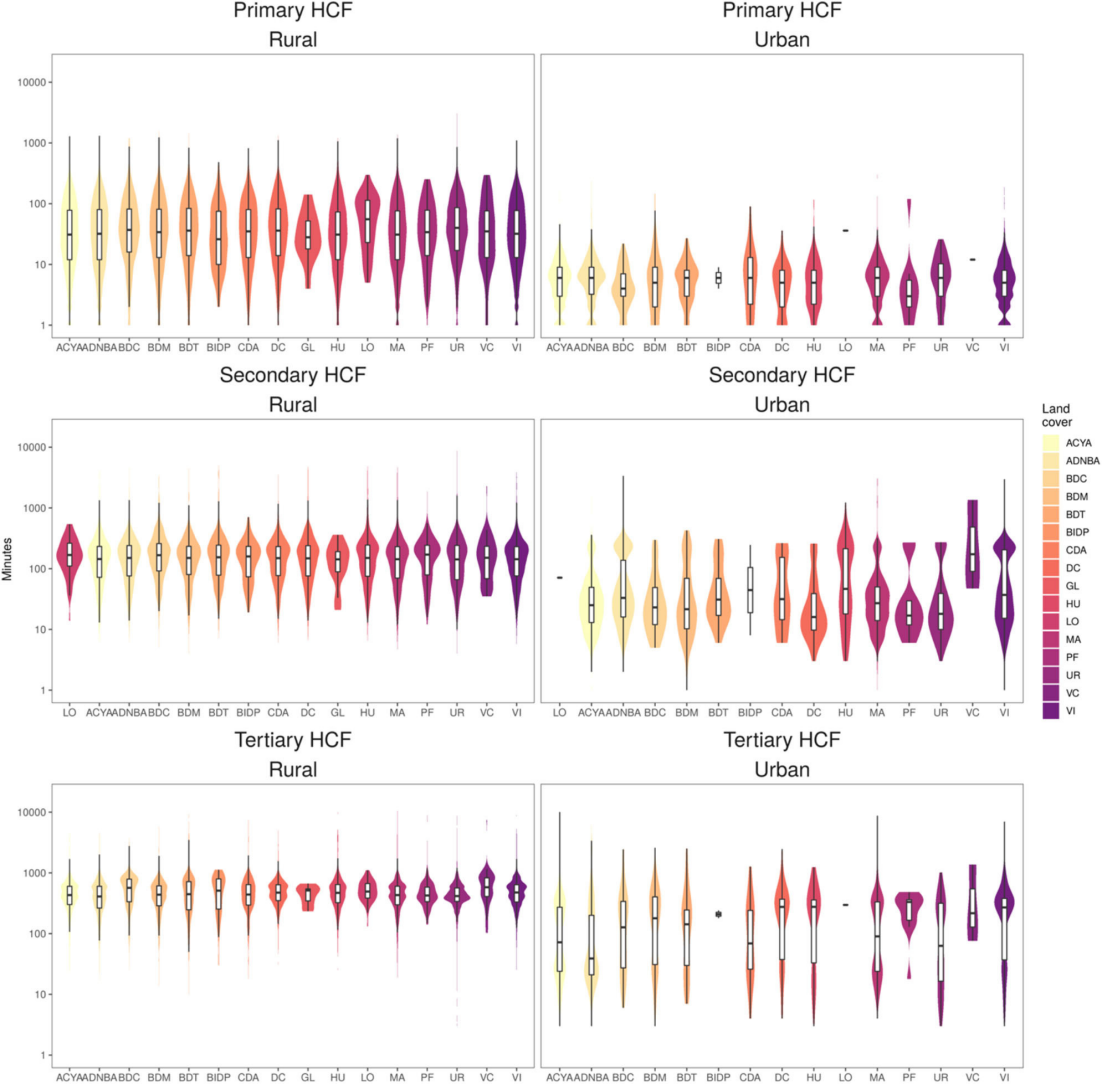
Distribution of travel time to most proximate health facility. Estimates across the 16 eco-regions land cover (*x*-axis) defined by the Peruvian Ministry of environment and rural/urban settings for primary, secondary and tertiary health care facilities (HCF). *Y*-axis in logarithmic scale.

High heterogeneity was observed in contrasting land coverage areas. The median travel time was 5.3-fold higher in rural (85 min; IQR = 11–7,819) than in urban settings (16 min; IQR = 11–835) to a primary HCF; 3.2-fold higher in rural (226 min; IQR = 11–12,429) than in urban settings (70 min; IQR = 11–3,386) to a secondary HCF; and 2.4-fold higher in rural (568 min; IQR = 11–35,753) than in urban settings (235 min; IQR = 11–10,048) to a tertiary HCF. A larger variation in travel time to primary HCF was observed in rural compared to urban areas, and conversely, a larger variation in travel time to tertiary HCF was observed in urban compared to rural areas (Figure 3). The district-level stratified travel times in Figure 2 show that there was also strong heterogeneity within major ecological regions. The north-east part of the Amazon Region, which borders with Colombia and Brazil, presented the largest country-wide travel times to the most proximate health facilities. The largest travel times to the most proximate HCF within the Highland Region was observed in the southern areas of the Andes, and on the coast was observed on the southern coast. Contrasting distributions of travel time to the most proximate health facility was observed between the 16 eco-regions defined by the MEnv (Figure 3).

### Travel Time to Health Facilities Relative to Socio-Economic and Health Metrics

**Figure 4:**
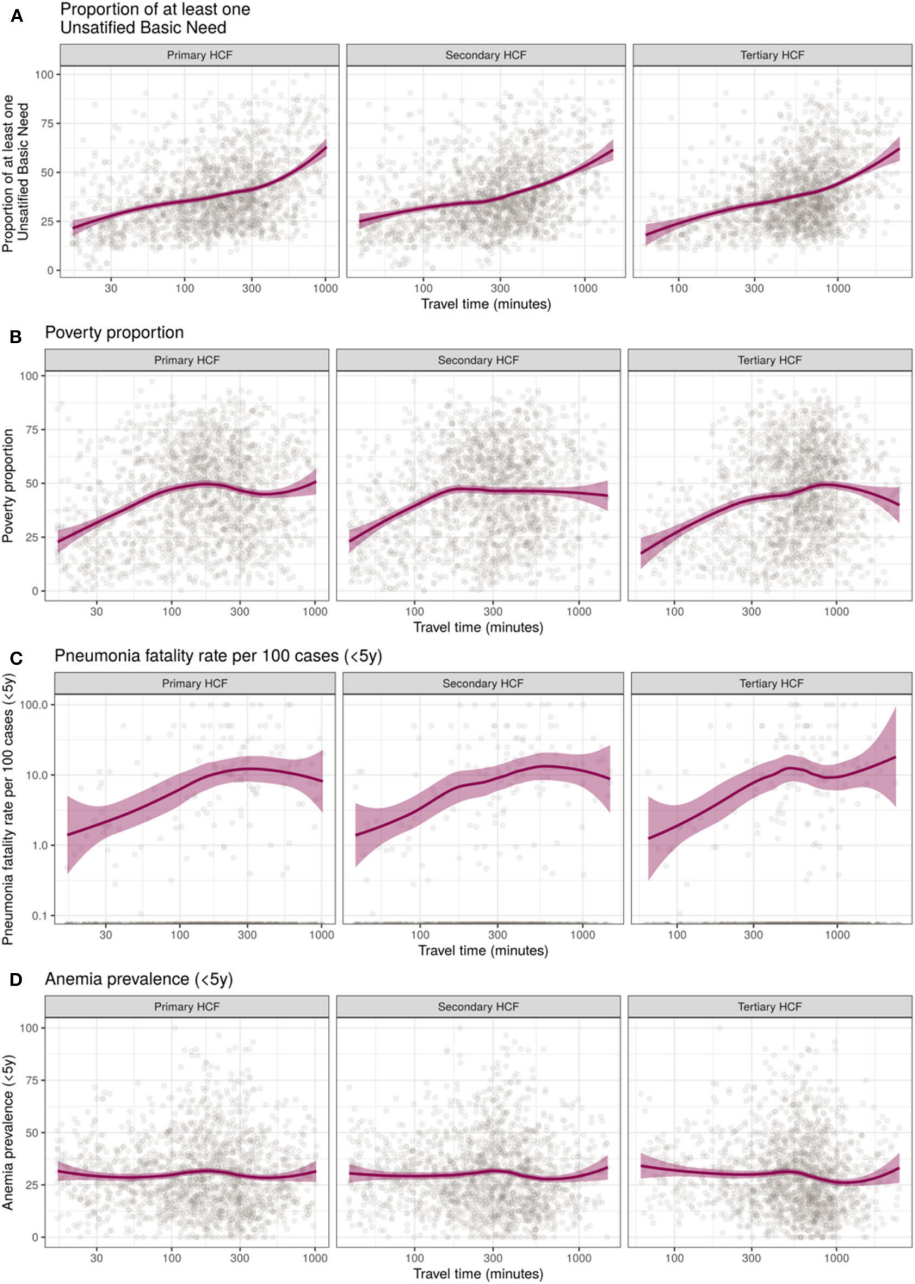
Trends of district-level median travel time to primary, secondary, and tertiary health care facilities (HCF) relative to socio-economic and health metrics in Perú. **(A)** Proportion of population with unsatisfied basic needs, **(B)** Poverty proportion, **(C)** Pneumonia fatality rate per 100 cases, and **(D)** Anemia prevalence in children under 5 years old. X-axis in logarithmic scale, locally estimated scatterplot smoothing (loess) trend line and 95% confidence intervals in purple.

In relation to the socio-economic metrics, a strong positive trend was observed between the travel time to HCF and the proportion of the population with at least one unsatisfied basic need (Figure 4A). This trend was consistent for primary, secondary, and tertiary HCF. Also, a positive yet less marked positive trend was observed in relation to the poverty proportion at the district level (Figure 4B).

Contrasting patterns were observed regarding the epidemiological metrics. An increased pneumonia fatality rate in children under 5 years was observed in districts with increased travel time to HCF. This trend seems to be more pronounced for increased travel times to tertiary HCF (Figure 4C). Conversely, no relation was observed between the travel time to primary, secondary, or tertiary HCF and the anemia prevalence in children between 6 months and 5 years old (Figure 4D).

## Discussion

This study explored the use of a novel cloud-based geospatial modeling approach fitted with detailed local geospatial data to accurately estimate the travel time to the most proximate HCF across a highly diverse geographical and ecological setting. Most of the variations in travel time to HCF arose from heterogeneous land coverage profiles and the contrast between urban and rural areas. This is particularly important due to the fact that in Peru and most low- and middle-income countries (LMIC), the most detailed data are available at a coarse administrative level that deters resource planning and health care provision in these countries. In addition, we explored the trends of travel time to HCF relative to socio-economics and epidemiological metrics. These trends showed a positive relation between large travel time estimates and underserved populations.

In settings with a scattered distribution of villages, timely access to health facilities is a cornerstone to improve the health status of impoverished populations and a first step to provide high-quality care (35, 36). Although the use of big data and high-detail datasets paves the way for a comprehensive quantification of geographical accessibility in terms of distance and travel time, these technologies were not previously applied to estimate geographical accessibility to health facilities until recently (21). Using this analytical approach, this study demonstrated that the population in the Jungle area have less accessibility since health care services are reachable at longer trajectories and travel time or less geographical accessibility. The dramatic heterogeneity in travel time to the most proximate health facility observed in this study corresponds to the contrasting landscape composition in the coast, highlands, and jungle regions. A dense road network was observed in the Coast, facilitating access to multiple services including health care as reported in other studies in India and Africa (6, 37). Conversely, sparse road coverage was observed in the Highlands, and only the two major cities in the Jungle region had roads.

Consistent with previous studies (13, 14), this study suggests heterogeneity in travel time to the most proximate health facility across areas with contrasting land coverage types. Despite this being widely accepted, few attempts have been made to quantify these heterogeneities. In addition, asymmetries were identified when the travel time to the most proximate HCF was compared along socio-economic profiles based on the unsatisfied basic needs proposed by the United Nations Development Programme (UNDP), and the poverty proportion was compared at the district level. Uneven trends of greater travel time to HCF (lower geographical accessibility) were observed among villages with higher rates of unmet basic needs. These results are consistent with previous reports of negative trends in geographical access to health care facilities in low-income populations (4, 38–40).

The implications of these results for the health care response were shown in the trends relative to epidemiological metrics. An important association with the pneumonia fatality rate in children under 5 years old was shown in this study representing a large burden of health effects due to low geographic accessibility. These findings are in accordance with previous studies in a variety of health outcomes (41–43). The lack of association between travel time to HCF and anemia prevalence observed in this study may be related to the interaction of many structural factors as described in previous studies (44, 45).

It is important to highlight that the analysis conducted in this study did not take into account variability due to climatic factors that may prevent displacement to health facilities (i.e., floods or landslides). However, Highlands and Jungle areas are more prone to these type of natural disasters, leading to a conservative estimation of travel time in these areas. Traffic, which may greatly influence the estimates in the large cities, was not considered in the analysis and potentially led to an underestimation of the travel time to health facilities. In addition, seasonal variability may greatly affect some displacement routes such as rivers; however, only navigable rivers were considered in this approach and the availability to displace through these rivers are less affected by seasonality. Another important consideration of the least-cost-path algorithm used in this analysis is that we infer the lowest travel time boundary to reach a health facility. This consideration relies on the assumption that the villagers opt for this route despite the cost and danger of the route in addition to its availability, as explained above.

In addition, the data reported here were generated at a meso-scale, with a spatial resolution of 30 m. At this scale and resolution, some important details could be lost and affect the travel time estimations. For instance, in some settings, the travel time might be increased due to meandering rivers or roads that follow the morphology of the terrain. The model assumes that transit flows in a direct manner, meaning that zigzagging routes may cause our approach to underestimate the real travel time to reach a health facility. Despite these possible shortcomings, the proposed approach provided conservative yet useful estimates of travel times to health facilities that are important for planning of prevention and control strategies for multiple health-related events. This approach demonstrates that curation and alignment of geospatial data from multiple governmental institutions are important for national decision-making. In addition, the use of mapping and modeling techniques and “big data” was recognized as critical for better planning (21, 46, 47); however, a remaining challenge is the implementation of these approaches into routine disease prevention and control programs (46, 47). Future studies may consider population-weighted estimates since rural areas mostly distant to HCF are areas with low a population density (22).

This study acknowledges the relevance of other components of health access that may play an important role in the underlying phenomena. The sole presence of clinic infrastructure does not assure proper health care delivery. The supply chain, human resources, financial accessibility, acceptability of services, and availability of treatment are some remaining barriers once geographical accessibility is overcome (3, 8, 48). Further studies were suggested to get a comprehensive understanding of the accessibility to health care in Peru and other LMICs.

## Conclusion

This study used a novel methodology to estimate the travel time to the most proximate health facilities to better understand and characterize the geographical accessibility profiles in Peru. Contrasting patterns were observed across heterogeneous land coverage areas and urban and rural settings and in relation to socio-economic and epidemiological metrics. These findings are important as first steps for tailoring strategies to deliver appropriate, affordable, and quality health care to impoverished populations.

## Data Availability Statement

All datasets generated for this study are included in the Supplementary Material and at https://figshare.com/articles/dataset/Travel_time_to_health_facilities_as_a_marker_ of_geographical_accessibility_across_heterogeneous_land_coverage_in_Peru/12894485.

## Author Contributions

GC-E and JM conceived, designed the study, and wrote the initial draft of the manuscript. GC-E, KT-L, and EM collected and analyzed the data. All authors reviewed and approved the final manuscript.

## Conflict of Interest

The authors declare that the research was conducted in the absence of any commercial or financial relationships that could be construed as a potential conflict of interest.
