# Supplementary material for: Travel Time to Health Facilities as a Marker of Geographical Accessibility Across Heterogeneous Land Coverage in Peru
Source: Front Public Health. 2020 Sep 16;8:498. doi: 10.3389/fpubh.2020.00498 (PMC7524891; doi:10.3389/fpubh.2020.00498)
Supplement: Supplementary file 1 [file Table_1.DOCX]

Travel time to health facilities as a marker of geographical accessibility across heterogeneous land coverage in Peru

**Gabriel Carrasco-Escobar^1,2,*^, Edgar Manrique^1^, Kelly Tello-Lizarraga^3^, J. Jaime Miranda^4,5^**

*** Corresponding Author:** Gabriel Carrasco Escobar, MSc, PhD(c): [gabriel.carrasco@upch.pe](about:blank)

^1^ Health Innovation Lab, Institute of Tropical Medicine “Alexander von Humboldt”, Universidad Peruana Cayetano Heredia, Lima, Peru

^2^ Division of Infectious Diseases, Department of Medicine, University of California San Diego, La Jolla, CA, USA

^3^ Facultad de Salud Publica y Administración, Universidad Peruana Cayetano Heredia, Lima, Peru

^4^ CRONICAS Centre of Excellence in Chronic Diseases, Universidad Peruana Cayetano Heredia, Lima, Peru.

^5^ School of Medicine, Universidad Peruana Cayetano Heredia, Lima, Peru

**Keywords:** Travel time, geographic accessibility, health care accesibility

**Supplementary Information 1:** Travel scenario

| **Category description** | **Speed (km/h)** | **Travel mode** |
| --- | --- | --- |
| **MODIS MCD12Q1 product – Land Cover type 1** | | |
| Evergreen Needleleaf Forests: dominated by evergreen conifer trees | 3.24 | Walking |
| Evergreen Broadleaf Forests: dominated by evergreen broadleaf and palmate trees | 1.62 | Walking |
| Deciduous Needleleaf Forests: dominated by deciduous needleleaf (larch) trees | 3.24 | Walking |
| Deciduous Broadleaf Forests: dominated by deciduous broadleaf tree | 4 | Walking |
| Mixed Forests: dominated by neither deciduous nor evergreen (40-60% of each) tree type | 3.24 | Walking |
| Closed Shrublands: dominated by woody perennials | 3 | Walking |
| Open Shrublands: dominated by woody perennials | 4.2 | Walking |
| Woody Savannas: tree cover 30-60% | 4.86 | Walking |
| Savannas: tree cover 10-30% | 4.86 | Walking |
| Grasslands: dominated by herbaceous annuals | 4.86 | Walking |
| Permanent Wetlands: permanently inundated lands with 30-60% water cover and >10% vegetated cover | 2 | Walking |
| Croplands: at least 60% of area is cultivated cropland | 2.5 | Walking |
| Urban and Built-up Lands: at least 30% impervious surface area including building materials, asphalt and vehicles | 5 | Walking |
| Cropland/Natural Vegetation Mosaics: mosaics of small-scale cultivation 40-60% with natural tree, shrub, or herbaceous vegetation | 3.24 | Walking |
| Permanent Snow and Ice: at least 60% of area is covered by snow and ice for at least 10 months of the year | 1.62 | Walking |
| Barren: at least 60% of area is non-vegetated barren (sand, rock, soil) areas with less than 10% vegetation | 3 | Walking |
| Water Bodies: at least 60% of area is covered by permanent water bodies | 1 | Boat |
| **Road Infrastructure** | | |
| National roads | 80 | Motorized |
| Departmental roads | 50 | Motorized |
| Vecinal roads | 30 | Motorized |
| **HydroSHEDS Flow Accumulation** | | |
| Navigable rivers | 9 | Boat |

**Supplementary Information 2.** List of complete and shortlist of ecosystems provided by the Ministry of Environment.

| **Shortlist** | **Original Category** |
| --- | --- |
| Coastal and andean agriculture | Coastal and andean agriculture |
| Non-Amazon forest area | Non-Amazon forest area |
| Non-Amazon forest area | Sand bank |
| Hill forest | Low hill forest with Shiringa |
| Hill forest | Low hill forest with bale |
| Hill forest | Dry hill forest |
| Hill forest | Low hill forest |
| Hill forest | High hill forest |
| Hill forest | Dry hill high forest |
| Hill forest | Dry hill forest with chesnut |
| Hill forest | high hill forest with bale |
| Hill forest | High hill forest of the Divisor |
| Mountain forest | Basimontano mountain forest |
| Mountain forest | Altimontano mountain forest |
| Mountain forest | Mountain forest |
| Mountain forest | Meander plain forest |
| Mountain forest | Dry mountain forest |
| Mountain forest | High relict Andean forest |
| Mountain forest | Inter-Andean relict forest |
| Mountain forest | Mountain forest |
| Mountain forest | Western Andean montane forest |
| Mountain forest | Mesoandino relict forest |
| Mountain forest | Mountain Palm Forest |
| Mountain forest | Mesoandine relict coniferous forest |
| Mountain forest | Mountain semi-deciduous forest |
| Terrace forest | High terrace forest with chestnut |
| Terrace forest | Riparian dry forest |
| Terrace forest | Blackwater Flooded Terrace Forest |
| Terrace forest | Low Terrace Forest |
| Terrace forest | Savanna dry forest |
| Terrace forest | High terrace forest |
| Terrace forest | Lomada dry forest |
| Terrace forest | Piedmont Dry Forest |
| Terrace forest | Low terrace forest with bale |
| Terrace forest | High terrace forest with bale |
| Terrace forest | Low terrace forest with chestnut |
| Flooded Palm Forest | Flooded Palm Forest |
| Flooded Palm Forest | Flooded basimontano palm forest |
| Water bodies | Lagoons, lakes and cochas |
| Water bodies | River |
| Water bodies | Dam |
| Coastal desert | Coastal desert |
| Glacier | Glacier |
| Wetland | Bofedal |
| Wetland | Coastal wetland |
| Wetland | Hydrophilic grassland |
| Wetland | Mangrove swamp |
| Wetland | Albufera |
| Hill | Hill |
| Shrub thicket | Shrub thicket |
| Shrub thicket | Shrub bush |
| Forest Plantation | Forest Plantation |
| Coastal vegetation | White sand vegetation |
| Coastal vegetation | Island vegetation |
| Coastal vegetation | Tillandsial |
| Interandean vegetation | Andean Pajonal |
| Interandean vegetation | Cardonal |
| Interandean vegetation | High Andean area with little and no vegetation |
| Interandean vegetation | Highland, desolate |
| Interandean vegetation | Jalca |
| Interandean vegetation | Interandean savanna |

**Supplementary Information 3**

Code GEE: [https://code.earthengine.google.com/1bf3524cfebcb89fa540e4c70b699c24](about:blank)

Output GEE: [https://edgarmanrique30.users.earthengine.app/view/country-wide-map-of-travel-time-to-health-facilities-for-2018](about:blank)
